# Syntheses of spliceostatins and thailanstatins: a review

**DOI:** 10.3762/bjoc.16.166

**Published:** 2020-08-13

**Authors:** William A Donaldson

**Affiliations:** 1 Department of Chemistry, Marquette University, P. O. Box 1881, Milwaukee, WI 53201-1881, USA

**Keywords:** antiproliferative, polyketide natural products, tetrahydropyrans, total synthesis

## Abstract

The spliceostatins/thailanstatins are a family of linear peptides/polyketides that inhibit pre-mRNA splicing, and as such act as potent cytotoxic compounds. These compounds generally contain 9 stereocenters spread over a common (2*Z*,4*S*)-4-acetoxy-2-butenamide fragment, an (all-*cis*)-2,3,5,6-tetrasubstituted tetrahydropyran fragment and a terminal oxane ring joined by a dienyl chain. Due to the impressive antitumor properties of these compounds, along with their complex structure, a number of total syntheses have been reported. This review will compare the synthetic strategies reported through the end of 2019.

## Introduction

**Figure 1:**
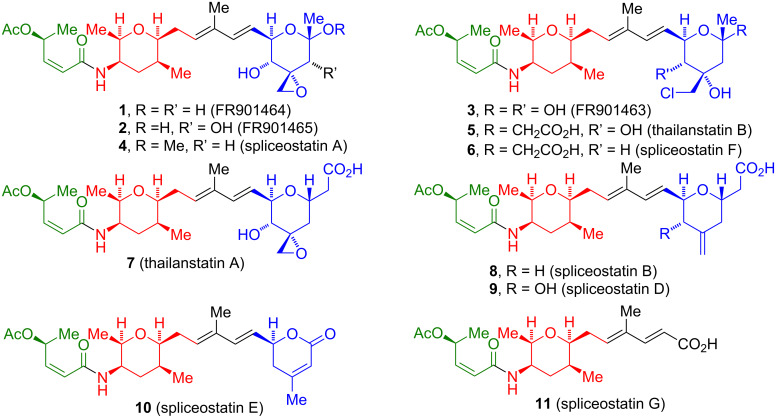
Structures of spliceostatins/thailanstatins.

The spliceostatins/thailanstatins (Figure 1) are a family of linear peptide/polyketide natural products isolated from the bacteria *Burkholderia* sp. FERM BP-3421 [1–3] (originally identified as *Pseudomonas* sp. No 2663) and *Burkholderia* sp. MSMB 43 [4–5]. These compounds are of interest due to their ability to bind to a subunit of the human spliceosome, splicing factor 3b [6], which inhibits pre-mRNA splicing, and as such act as potent cytotoxic compounds. A review of the discovery, target identification, and biological applications of the compounds that exhibit these binding characteristics has been published [7]. These compounds all contain a common (2*Z*,4*S*)-4-acetoxy-2-butenamide fragment (in green, Figure 1), appended to an (all-*cis*)-2,3,5,6-tetrasubstituted tetrahydropyran fragment (in red, Figure 1). The members of this family primarily differ with respect to the terminal oxane ring (in blue, Figure 1) which is attached to the common fragments by a dienyl chain. The exciting antitumor properties of these compounds (Table 1), along with their complex structure, have led to a significant synthetic activity. The present review will cover the total syntheses of the spliceostatins/thailanstatins through the end of 2019.

**Table 1:** Biological activities of spliceostatins/thailanstatins (IC_50_ values in nM).

	tumor cell line				
	
compound	MCF-7	A549	HCT116	SW480	P388

FR901464 (**1**)^a^	0.91	0.66	0.31	0.51	1.69
FR901465 (**2**)^a^	0.59	0.44	0.34	0.53	0.48
FR901463 (**3**)^a^	0.46	0.35	0.22	0.40	0.82

	H1975	N87	BT474	MDA-MB-DYT2	MDA-MB-468

thailanstatin B (**5**)^b^	n.t.^c^	>100	>100	n.t.	>100
spliceostatin F (**6**) ^b^	n.t.	0.641	1.85	n.t.	1.35
thailanstatin A (**7**) ^b^	320	59	145	161	142
spliceostatin B (**8**) ^b^	30	n.t.	n.t.	n.t.	n.t.
spliceostatin D (**9**) ^b^	950	n.t.	n.t.	n.t.	n.t.
spliceostatin E (**10**)^b^	n.t.	3.67	3.72	4.16	1.56
spliceostatin G (**11**)^b^	n.t.	>100	>100	>100	n.t.

^a^See reference [1]. ^b^See reference [5]. ^c^n.t. = not tested

## Review

### Synthesis of the (2*Z*,4*S*)-4-acetoxy- or protected (2*Z*,4*S*)-4-hydroxy-2-butenoic acid fragment

**Scheme 1:**
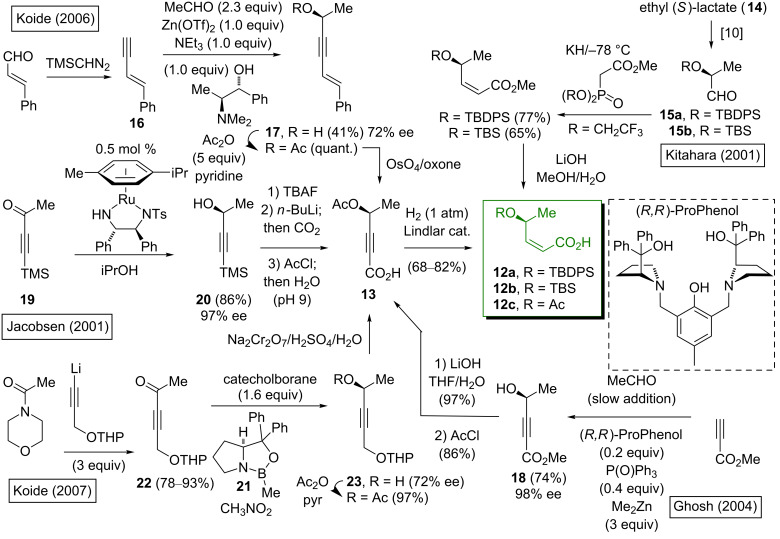
Synthetic routes to protected (2*Z*,4*S*)-4-hydroxy-2-butenoic acid fragments.

The (2*Z*,4*S*)-4-acetoxy-2-butenoic acid fragment is common to all of the spliceostatins/thailanstatins. The various routes to this building block (Scheme 1) serve as a tutorial on the methodology for the asymmetric synthesis of a (*Z*)-2-hydroxy-3-pentene unit, with most proceeding via the *cis*-reduction of 4-acetoxy-2-pentynoic acid (**13**). The synthesis by Kitahara et al. [8–9] is the exception, where the chiral center is derived from relatively inexpensive (*S*)-ethyl lactate (**14**). This was transformed into the silyl ether-protected 2-hydroxypropanals **15a** and **15b** via literature procedures [10], followed by the application of the Still–Gennari *Z*-selective Horner–Wadsworth–Emmons olefination [11]. Koide’s group [12–13] reported that the asymmetric addition of the enyne **16** to acetaldehyde in the presence of zinc triflate and the chiral additive *N*-methylephedrine [14] gave the (*S*)-propargyl alcohol **17** in 72% ee; the chiral purity could be improved to 96% ee by recrystallization. Unfortunately, attempts to catalyze this reaction with Zn(OTf)_2_/ephedrine were unsuccessful. More recently, Ghosh and co-workers [15] used the (*R,R*)-ProPhenol ligand [16] to accomplish a catalytic asymmetric addition of methyl propynoate to acetaldehyde to give **18** in high enantiopurity (98% ee). Jacobsen’s group [17–18] utilized the Noyori Ru-catalyzed transfer hydrogenation [19] of the 3-butyne-2-one **19**, which gave **20** with 97% ee. The removal of the silyl protecting group, followed by a carboxylation and acylation gave **13**. Koide’s group [13] reported a second-generation route to **13**, which utilized the Corey–Bakshi–Shibata chiral oxazaborolidine catalyst **21** [20] for the asymmetric reduction of the THP-protected 5-hydroxy-3-pentyn-2-one **22** to generate the secondary alcohol **23**. The acylation of **23**, followed by the treatment with Jones’ reagent effected the THP deprotection as well as an overoxidation to give **13**. The *syn*-selective reduction of **13** was accomplished with a balloon worth of pressure of H_2_ in the presence of the Lindlar catalyst. Of the asymmetric strategies, Jacobsen’s and Ghosh’s routes proceeded with the highest enantioselectivity; the catalyst loading was lower for the Jacobsen route (0.5 mol %) compared to the Ghosh route (20 mol %).

### Synthesis of the (all-*cis*)-2,3,4,5-tetrasubstituted tetrahydropyran fragment

#### Syntheses from ʟ-threonine-derived aldehyde

**Scheme 2:**
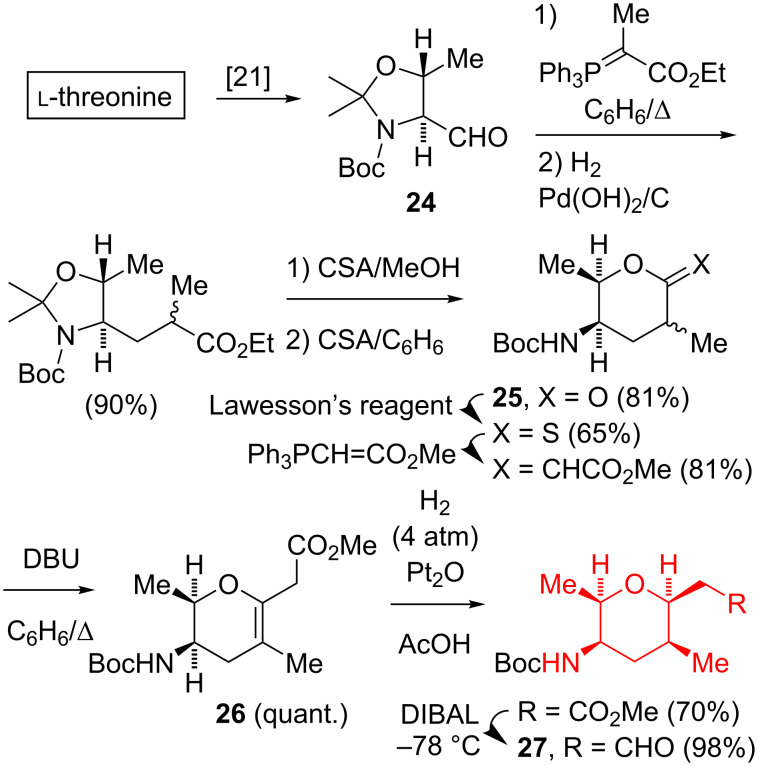
Kitahara synthesis of the (all-*cis*)-2,3,5,6-tetrasubstituted tetrahydropyran.

Three groups utilized 4-formyl-2,2,5-trimethyl-3-oxazolidine (**24**) [21], derived from relatively inexpensive ʟ-threonine (<$1/g in bulk) as a chiral pool precursor for the amine stereocenter of the (all-*cis*)-2,3,5,6-tetrasubstituted tetrahydropyran fragment. In Kitahara’s synthesis [8–9], the Wittig olefination of **24**, followed by a catalytic hydrogenation, removal of the dimethylaminal protecting group, and lactonization gave **25** as a mixture of diastereomers (Scheme 2). The further transformation of **25** afforded the dihydropyran **26**, which upon catalytic hydrogenation over Pt_2_O and then low-temperature DIBAL reduction afforded the all-*cis* tetrasubstituted tetrahydropyran **27**.

**Scheme 3:**
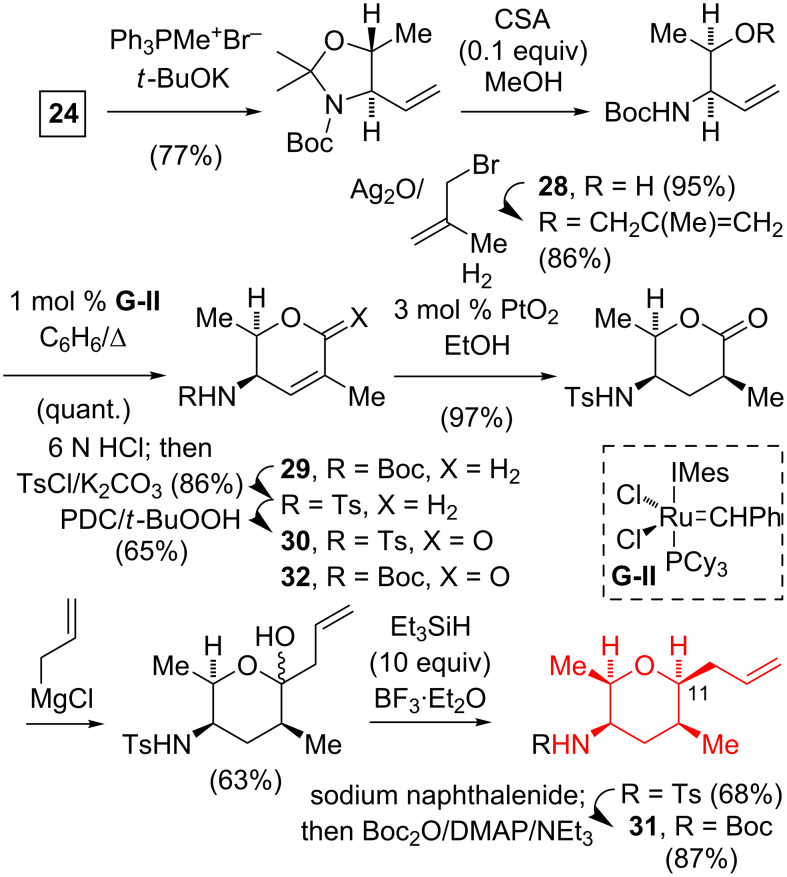
Koide synthesis of (all-*cis*)-2,3,5,6-tetrasubstituted tetrahydropyran.

In Koide et al.’s synthesis, the unsaturated lactone **29** was prepared via the Wittig methenylation of **24**, followed by aminal hydrolysis, methallylation of the 2° alcohol **28**, and ring-closing metathesis using Grubbs’ 2nd generation catalyst (**G-II**, Scheme 3) [13]. Replacing the *N*-Boc protecting group with an *N*-tosyl group and allylic oxidation gave **30**. The introduction of the allyl group at C-11 made use of the Kishi protocol [22] of the allyl-Grignard addition, followed by an ionic reduction. The *N*-tosyl group was removed and the resultant amine protected to give the *N*-Boc tetrahydropyran **31**. The Koide group had originally attempted the reduction–allylation–ionic reduction sequence on the Boc-protected amine **32**, however, this gave lower overall yields due to the competing formation of a pyrrolidine byproduct.

Using the Stork–Zhao conditions [23], the Nicolaou group performed an α-methyliodomethylenation of **24** to give **33** with a good stereoselectivity (*Z:E* ≈ 95:5, Scheme 4) [24–25]. After the hydrolysis of the dimethylaminal and the reaction of the resultant amine with phthalic anhydride, a Pd-catalyzed Stille coupling [26] of **34** with 3-(tributylstannyl)-2-propen-1-ol gave **35**. The oxidation of **35** with an excess of MnO_2_ gave the (2*E*,4*Z*)-dienal **36**. Alternatively, the Stille coupling of **34** with 3-(tributylstannyl)acrolein also afforded **36**. After much experimentation with up to eight amine catalysts, this group found that the intramolecular oxa-Michael addition using 20 mol % of the diarylprolinol organocatalyst **37** in the presence of benzoic acid gave **38**. The use of the enantiomer of **37** gave the dihydropyran with the opposite configuration at C-11. The catalytic hydrogenation of **38** proceeded with a poor stereoselectivity, however, a similar reduction of the diethyl acetal of **38**, followed by an acetal hydrolysis gave the aldehyde **39** with a good stereocontrol (>10:1).

**Scheme 4:**
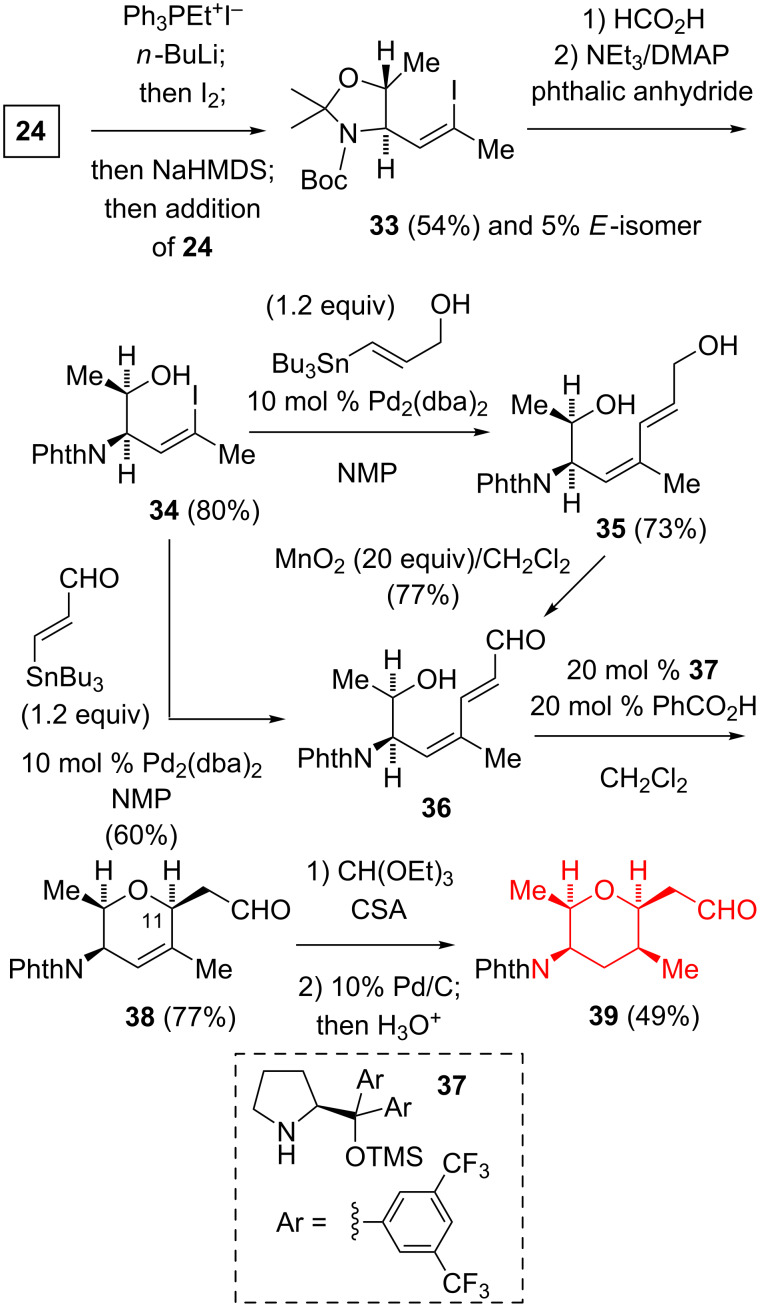
Nicolaou synthesis of the (all-*cis*)-2,3,5,6-tetrasubstituted tetrahydropyran.

It is not possible to make a direct comparison of the efficiency of these three routes as they do not lead to an identical endpoint. However, Nicolaou’s route is the shortest (6 or 7 steps, 9.8–9.2% yield), while Kitahara’s synthesis is the highest-yielding and does not involve the use of expensive transition metals or organocatalysts.

#### Syntheses to generate the C-14 stereocenter via C–N bond formation

**Scheme 5:**
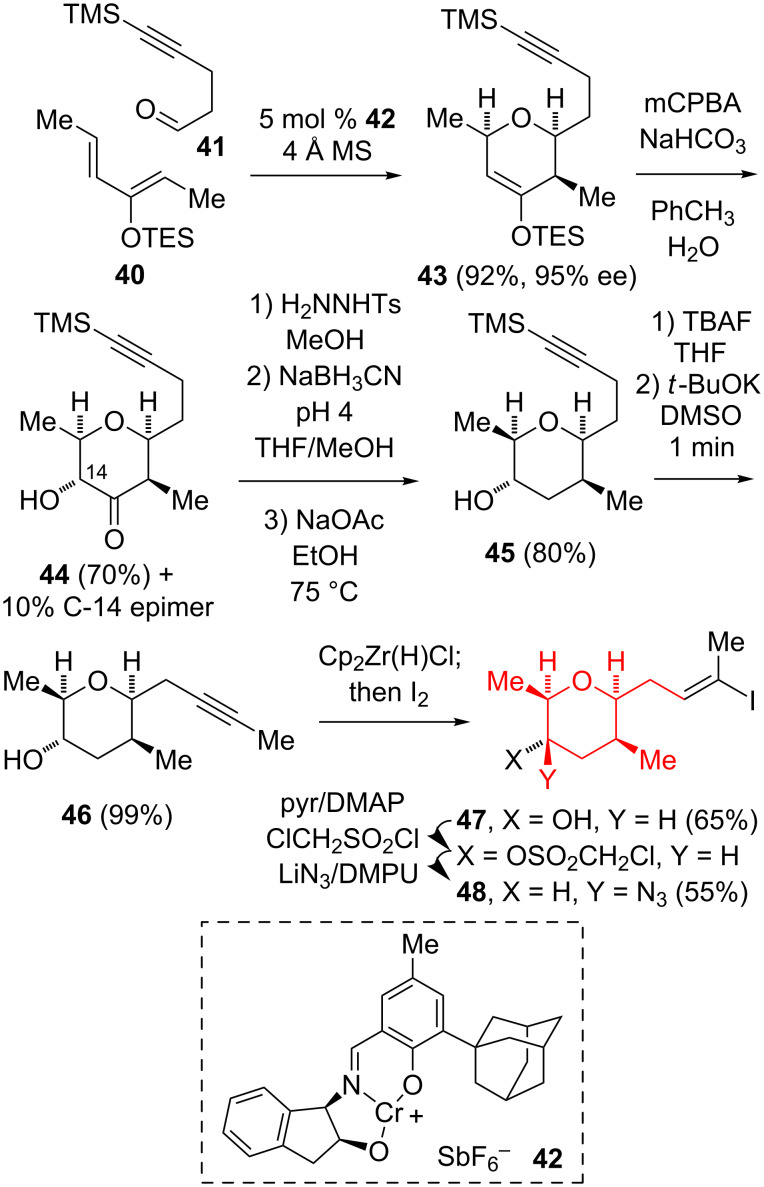
Jacobsen synthesis of the (all-*cis*)-2,3,5,6-tetrasubstituted tetrahydropyran.

Two groups implemented strategies that rely on the generation of the C-14 stereocenter by stereoselective C–N bond formation. The Jacobsen group utilized an asymmetric Cr(III)-catalyzed cycloaddition reaction [27] between (2*Z*,4*E*)-3-(triethylsilyloxy)-2,4-hexadiene (**40**) and the aldehyde **41** to generate the 4-silyloxydihydropyran **43** in a high yield and enantioselectivity (Scheme 5) [17–18]. The Rubottom oxidation [28] of **43** gave a separable mixture of the desired **44** and its C-14 epimer (≈7:1 ratio). The reductive deoxygenation of **44** proceeded via the tosylhydrazone to afford **45**, which upon desilylation and alkyne isomerization produced **46**. The hydrozirconation of **46** with Schwartz’s reagent under equilibrating conditions, followed by the reaction with I_2_ gave the vinyl iodide **47**. Finally, the activation of the C-14 hydroxy group and the S_N_2 displacement with azide gave the C-8–C-16 fragment **48**.

**Scheme 6:**
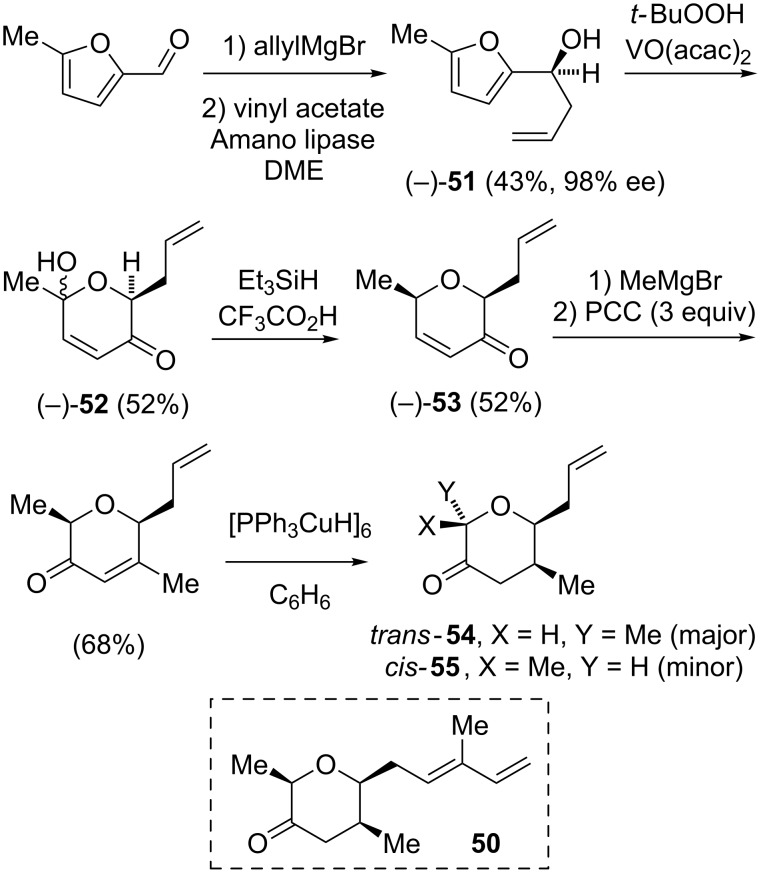
Unproductive attempt to generate the (all-*cis*)-tetrahydropyranone **50**.

Ghosh relied on a reductive amination of the tetrahydropyranone **50** to generate the (all-*cis*)-tetrahydropyran fragment. This group reported multiple different routes to **50**. In an abortive route, the addition of allyl-Grignard to 5-methylfurfural, followed by the resolution of the racemic homoallylic alcohol with Amano lipase gave (−)-**51** (Scheme 6) [16]. An Achmatowicz oxidation [29] with *tert*-butyl hydroperoxide catalyzed by VO(acac)_2_ afforded the hemiketal **52**, and the ionic reduction [22] of **52** gave the enone (−)-**53**. The enone transposition of **53** was effected by the addition of methyl-Grignard, followed by the oxidation with PCC. Unfortunately, all attempted 1,4-reduction conditions afforded an inseparable mixture of *trans*-**54** as the major product along with minor amounts of the desired *cis*-**55**.

**Scheme 7:**
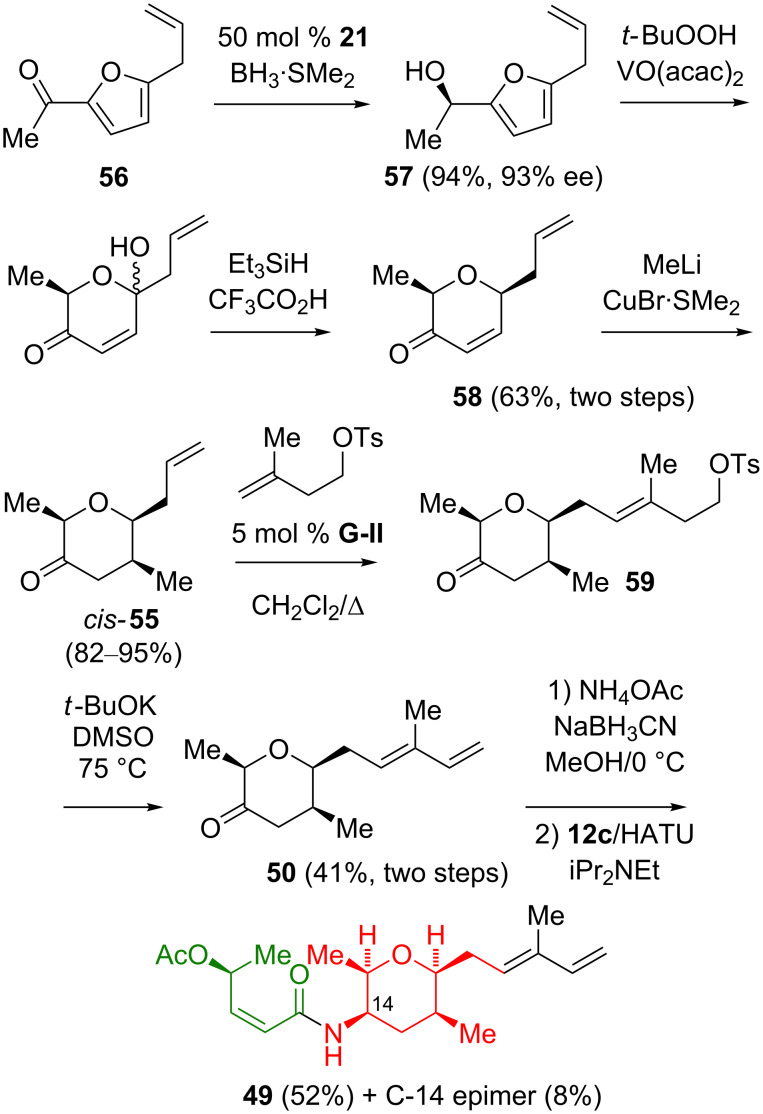
Ghosh synthesis of the C-7–C-14 (all-*cis*)-tetrahydropyran segment.

Alternatively, the asymmetric reduction of the 2-acylfuran **56** using the Corey–Bakshi–Shibata reagent (**21**) [30] gave the alcohol **57** in a high yield and high enantioselectivity (Scheme 7) [15–16]. The Achmatowicz oxidation and ionic reduction generated the enone **58**, which is regioisomeric with **53**. The 1,4-addition of lithium dimethylcopper gave the desired *cis*-**55** with high a diastereoselectivity (25:1). The cross-metathesis of **55** with 3-methyl-3-buten-1-yl tosylate in the presence of Grubbs’ 2nd generation catalyst yielded **59**, which, upon elimination with potassium *tert*-butoxide led to the diene **50**. The reductive amination of **50** afforded an inseparable mixture of the C-14 amines (6:1 ratio). However, the amidation of this mixture with **12c** gave a separable mixture of the desired **49** (52%) along with the C-14 epimer (8%).

**Scheme 8:**
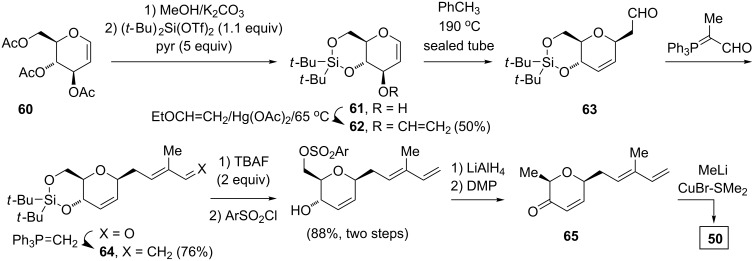
Ghosh’s alternative route to the (all-*cis*)-tetrahydropyranone **50**.

More recently, the Ghosh group provided an alternative synthesis from the triacetyl ᴅ-glucal **60** (Scheme 8) [31]. The exhaustive hydrolysis and selective protection as the 4,6-*O*-di-*tert*-butylsilylene derivative **61** [32] was followed by a 3-*O*-vinylation. A thermal 3,3-sigmatropic Claisen rearrangement of **62** gave the *cis*-2,6-disubstituted dihydropyran **63**, which upon sequential Wittig olefination with 2-(triphenylphosporanylidene)propanal and then methylenetriphenylphosphorane yielded the diene **64**. The removal of the di-*tert*-butylsilylene protecting group and the selective arylsulfonylation of the primary alcohol was effected with the bulky 2,4,6-triisopropylsulfonyl chloride, which upon reduction with aluminum hydride, followed by the oxidation of the remaining alcohol group gave the dihydropyran-3-one **65**. In a fashion similar to that of the dihydropyranone **58** [16], the 1,4-addition of dimethyl copper lithium to **65** gave the desired *cis*-**50** as a single diastereomer [31], allowing for a convergence with the route in Scheme 7.

**Scheme 9:**
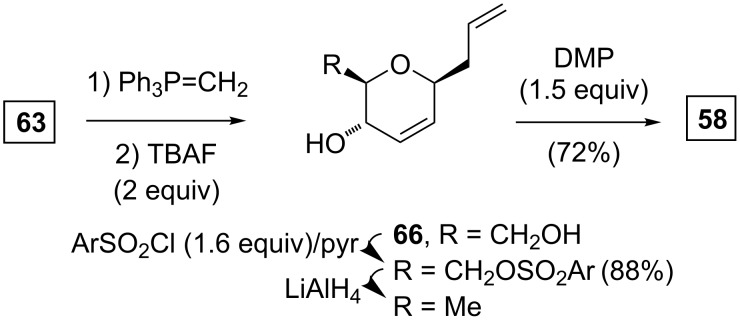
Alternative synthesis of the dihydro-3-pyrone **58**.

Alternatively, the Wittig methenylation of **63**, followed by a silyl ether cleavage gave **66** (Scheme 9) [33]. A three-step sequence similar to that from **64** to **65** allowed for the transformation of **66** to **58**, and thus providing an additional pathway for a convergence.

### Synthesis of the C-1–C-6 tetrahydropyran fragment of FR901464 (**1**)/spliceostatins/thailanstatins

#### Syntheses of the C-1–C-6 segment of FR901464 (**1**) from chiral pool precursors

**Scheme 10:**
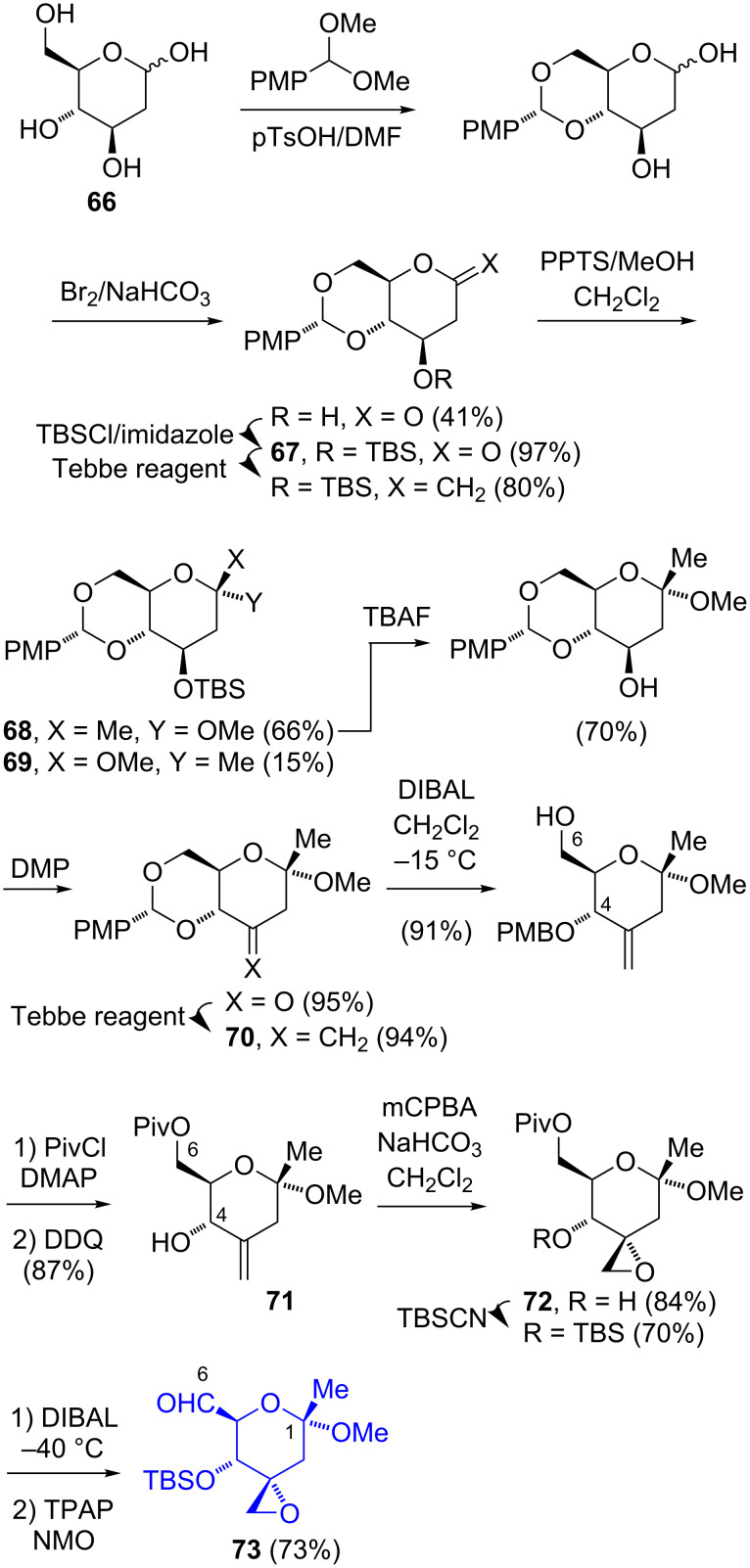
Kitahara’s 1st-generation synthesis of the C-1–C-6 fragment of FR901464 (**1**).

Kitahara’s group fashioned the C-1–C-6 tetrahydropyran fragment of FR901464 (**1**) from commercially available 2-deoxy-ᴅ-glucose. In the first-generation approach (**66**, Scheme 10) [8], a sequence of protection and oxidation steps generated the tetrahydropyrone **67** at which point an olefination with the Tebbe reagent and the addition of methanol afforded the cyclic ketal as a separable mixture of the diastereomers **68** and **69** (≈4.5:1 ratio). The desilylation of **68** gave the 2° alcohol, which after oxidation and Tebbe olefination afforded the exocyclic olefin **70**. The manipulation of the C-4 and C-6 protecting groups gave the secondary allylic alcohol **71**, which underwent an epoxidation with mCPBA to give **72**. A second sequence of C-4/C-6 protection, manipulation, and oxidation gave the aldehyde **73**. The disadvantages of this route include the overall length (13 or 14 steps), low yield (5.4%), and the relative expense of the starting material ($22.3/g).

**Scheme 11:**
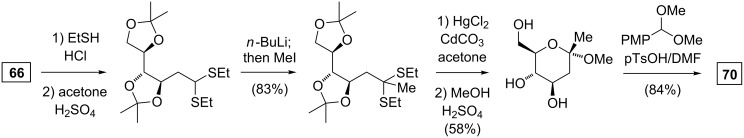
Kitahara 1st-generation synthesis of the C-1–C-6 fragment of FR901464 (**1**).

In their second-generation approach (Scheme 11) [9], the acetonide-protected dithiane was alkylated according to Horton’s procedure [34]. A deprotection and cyclic-ketal formation gave **70**, allowing for a convergence with Scheme 10.

**Scheme 12:**
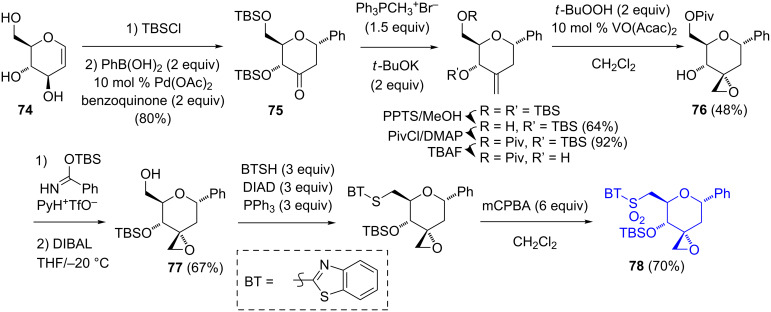
Nimura/Arisawa synthesis of the C-1-phenyl segment.

Very recently, the groups of Nimura and Arisawa reported the synthesis of a phenyl C-glucoside derivative of spliceostatin beginning from ᴅ-glucal (Scheme 12) [35]. A Heck coupling of the tris(trimethylsilyl) ether of **74** with phenylboronic acid in the presence of Pd(OAc)_2_ and benzoquinone (BQ) gave the *C*-phenyl glucoside **75** [36]. Notably, the use of oxidants other than BQ gave either the TMS enol ether or the 2,3-dihydro-6-phenyl-4*H*-pyran-4-one. The C-3 exocyclic methylene group was introduced by a Wittig olefination, and after the manipulation of the protecting groups, a VO(acac)_2_-catalyzed oxidation stereoselectively generated the spirocyclic epoxide **76**. A second-protecting group shuffle afforded the primary alcohol **77**. A Mitsunobu substitution of **77** with 2-mercaptobenzothiazole, followed by an oxidation with a large excess of mCPBA afforded the sulfone **78**. While not producing the identical product, this route is shorter and higher-yielding (11 steps, 11.5%) than the Kitahara synthesis. However, it suffers from repeated protection/deprotection steps.

**Scheme 13:**
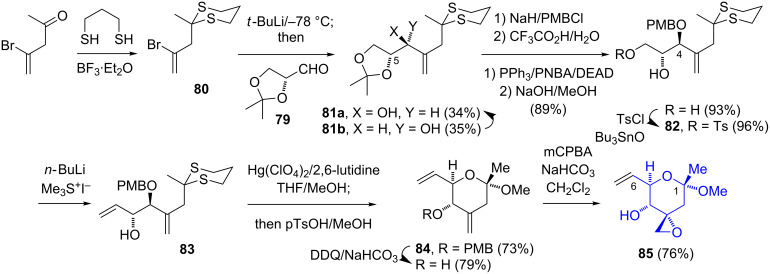
Ghosh synthesis of the C-1–C-6 fragment of FR901464 (**1**) from (*R*)-glyceraldehyde acetonide.

The Ghosh group utilized (*R*)-glyceraldehyde acetonide (**79**, readily available from ᴅ-mannitol) as a chiral pool precursor for the introduction of the C-6 stereocenter (Scheme 13) [15–16]. The administration of the vinyllithium reagent, generated from the addition of the vinyl bromide **80** to **79**, gave a separable 1:1 mixture of the diastereomeric alcohols **81a** and **81b**. The undesired stereoisomer **81b** was converted into **81a** by a Mitsunobu reaction/hydrolysis sequence. The use of the dithiane **80** protecting group was crucial for the following steps. Initial attempts of using a dioxolane protecting group for the C-1 ketone (instead of dithiane) led to an insurmountable difficulty of the selective hydrolysis of the dioxolane and acetonide ketals. The protection of the C-4 hydroxy group, hydrolysis of the acetonide, and selective tosylation of the 1° alcohol were prerequisites for the generation of the C-5–C-6 bond. To this end, the reaction of **82** with the ylide generated from trimethylsulfonium iodide gave the allylic alcohol **83**. The removal of the dithiane protecting group and the cyclic-ketal formation gave **84**. The oxidative hydrolysis of the PMB ether and the reaction with mCPBA afforded the epoxide **85**. While relatively short (9 steps), the nonstereoselective formation of **81a**/**b** led to a lower overall yield.

#### Syntheses of the C-1–C-6 segment of FR901464 (**1**) via asymmetric catalysis

**Scheme 14:**
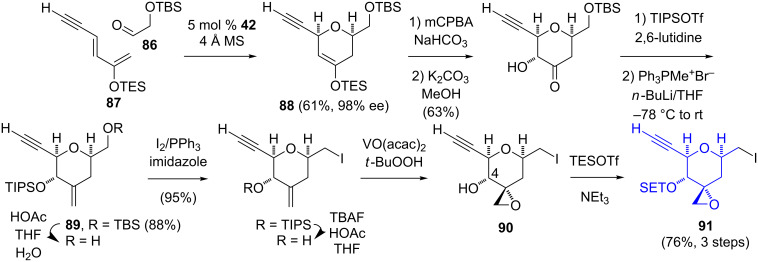
Jacobsen synthesis of the C-1–C-7 segment of FR901464 (**1**).

Similar to their preparation of the C-8–C-15 segment, Jacobsen’s group relied on a Cr(III)-catalyzed silyloxydiene/aldehyde cycloaddition for the C-1–C-7 segment (Scheme 14) [17–18]. The reaction of the protected glycolaldehyde **86** with (1*E*,3*E*)-2-triethylsilyloxy-1,3-hexadien-5-yne (**87**) in the presence of **42** gave the dihydropyran **88** with excellent enantioselectivity. A Rubottom oxidation, protection of the C-4 alcohol, and a Wittig methenylation afforded **89**. The selective deprotection of the primary TBS ether, followed by an Appel iodination and the cleavage of the secondary triisopropylsilyl ether were prerequisites for a vanadium-catalyzed stereoselective epoxidation of the exocyclic double bond to give **90**. The C-4 hydroxy group was eventually protected as a triethylsilyl ether. Through abortive attempts, Jacobsen found that the generation of the exocyclic epoxide prior to the formation of the C-6–C-9 conjugated diene was necessary in order to avoid the unwanted epoxidation of the C-6–C-7 olefin.

**Scheme 15:**
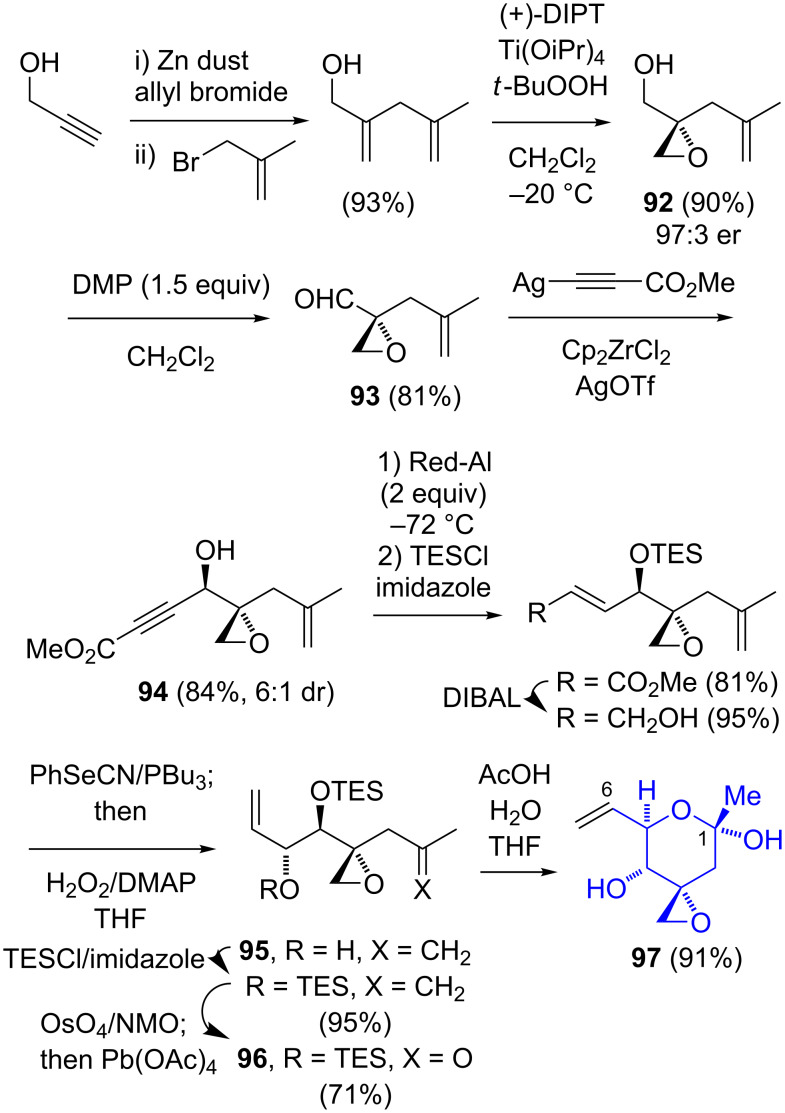
Koide synthesis of the C-1–C-7 segment of FR901464 (**1**).

Koide employed a unique strategy in which the exocyclic epoxide was generated as the initial stereocenter (Scheme 15) [12–13]. The Sharpless asymmetric epoxidation [37] of 5-methyl-2-methylene-4-penten-1-ol gave the epoxyalcohol **92** in 94% ee, which was oxidized to the aldehyde **93**. While the addition of the lithium salt of methyl propynoate proceeded in a nondiastereoselective fashion, the use of a zirconium/silver-mediated alkynylation gave the alcohol **94** with a 6:1 diastereoselectivity. The Red-Al reduction of **94**, protection of the 2° alcohol, and reduction of the enoate to an allylic alcohol were prerequisites for the stereoselective 2,3-sigmatropic selenoxide rearrangement to generate **95**. Further protecting group manipulation and Johnson–Lemieux cleavage afforded the methyl ketone **96**. The cyclic hemiketal **97** was unstable at an elevated temperature (37 °C), with *t*_1/2_ = 48 h. Both the Jacobsen (10 steps, 24.4% yield) and the Koide syntheses (11 steps, 22.3% yield) are relatively efficient in terms of the length and overall yield.

#### Syntheses of the C-1–C-5 segment of thailanstatin methyl esters

**Scheme 16:**
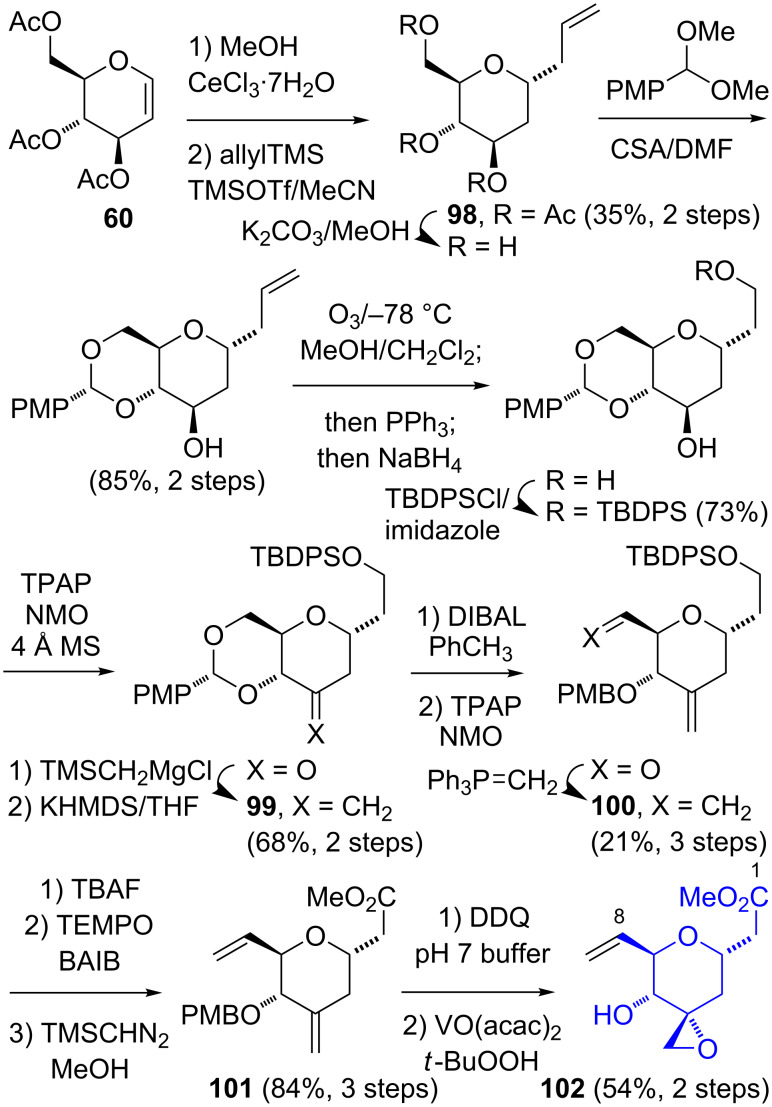
Ghosh synthesis of the C-1–C-5 segment **102** of thailanstatin A (**7**).

Both syntheses of the C-1–C-5 tetrahydropyran segment of thailanstatin A (**7**) utilize sugar precursors. In the Ghosh synthesis [31], the triacetyl ᴅ-glucal **60** is converted into the *trans* C-1-allylated tetrahydropyran **98** according to literature procedures [38–39] (Scheme 16). After a protecting group manipulation, the C-1 allyl group was truncated via ozonolysis, and the C-3 hydroxy group was transformed into an exocyclic methylene group to give **99**. The conversion of the C-6 alcohol moiety to a vinyl group by oxidation and Wittig methenylation to give **100** was relatively inefficient (31%) and was the main factor in diminishing the overall yield. The transformation of the silyl-protected 1° alcohol group of **100** into an ester afforded **101**. Finally, the C-3 spirocyclic oxirane was introduced stereoselectively by a VO(acac)_2_-directed epoxidation. While highly stereoselective, this relatively lengthy route was somewhat inefficient (16 steps, 1.4% overall yield).

**Scheme 17:**
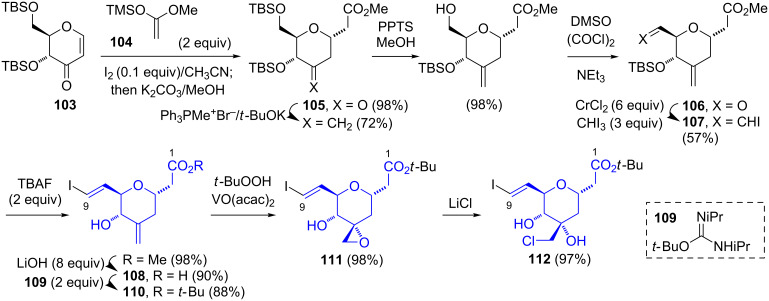
Nicolaou synthesis of the C-1–C-9 segments of spliceostatin D (**9**) and thailanstatins A (**7**) and B (**5**).

The Nicolaou synthesis [24–25] of the C-1–C-9 tetrahydropyranyl segment commenced with the protected dihydropyranone **103** (Scheme 17), readily prepared from ᴅ-glucal [38]. The application of an iodine-catalyzed Mukiyama–Michael addition of the ketene silyl acetal **104** to **103** afforded the *trans*-1,5-disubstituted tetrahydropyranone **105**. After the generation of the C-3-exocyclic olefin and functional group manipulation, the Takai olefination [40] of the aldehyde **106** gave the *trans*-vinyl iodide **107**. The removal of the silyl protecting group and hydrolysis of the methyl ester afforded the carboxylic acid **108**. A subsequently attempted Suzuki–Miyaura coupling of **108** with vinyl boronates was described as “capricious”, and thus the acid was esterified with the *tert*-butyl donor reagent **109** to afford **110**. In a fashion similar to that used by Ghosh’s group, the VO(acac)_2_-catalyzed epoxidation of **110** afforded the spirocyclic oxirane **111**. A subsequent ring opening with LiCl gave **112**, which was used in the synthesis of thailanstatin B (**5**). Nicolaou’s route to **111** (9 steps, 29.9% yield) is shorter and considerably more efficient than the Ghosh synthesis of **102**.

#### Syntheses of the C-1–C-6 segment of spliceostatin E (**10**)

**Scheme 18:**
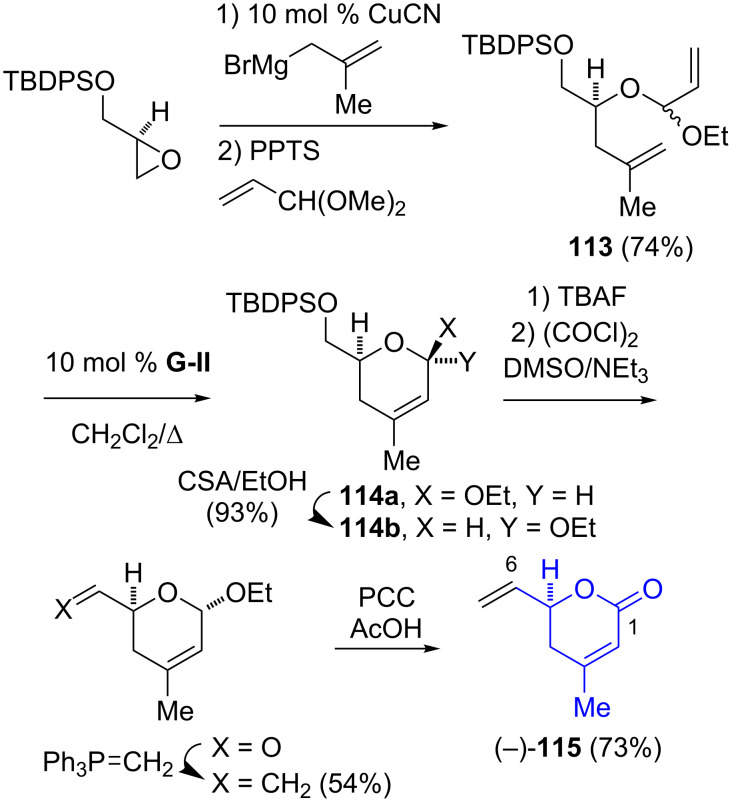
Ghosh synthesis of the C-1–C-6 segment **115** of spliceostatin E (**10**).

The Ghosh group’s synthesis of the C-1–C-6 segment of spliceostatin E (**10**) relied on a Cu-catalyzed Grignard addition to *tert*-butyldiphenylsilyl-protected (*R*)-glycidol, followed by the generation of the mixed acetal **113** (Scheme 18) [33]. A ring-closing metathesis gave an inseparable mixture of the dihydropyranyl ethers **114a** and **14b**, which could be equilibrated under acidic conditions (**114b**/**114a** > 20:1). A standard functional group manipulation afforded the vinyldihydropyran-2-one (−)-**115**.

### Fragment coupling to complete the synthesis of FR901464 (**1**)/spliceostatins/thailanstatins

#### Fragment coupling via Wittig and Julia olefinations

**Scheme 19:**
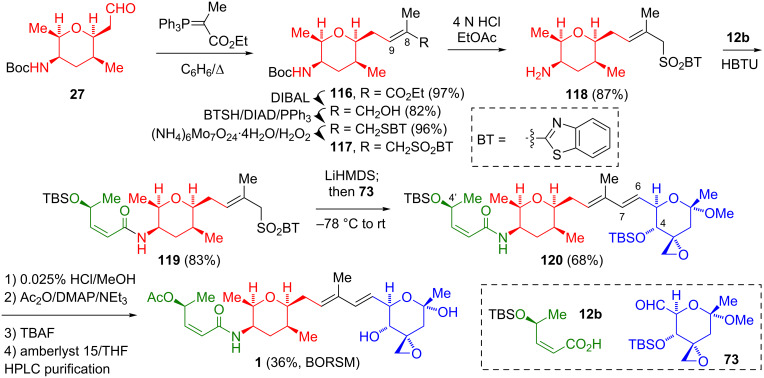
Fragment coupling via Wittig and modified Julia olefinations by Kitahara.

Kitahara’s group utilized Wittig and modified/one-pot Julia olefination [41] reactions to fashion the dienyl segment, joining the two tetrahydropyran fragments. As their 2nd-generation synthesis was more efficient, this will be described (Scheme 19) [9]. To this end, the C-9–C-15 aldehyde **27** underwent an olefination with (carbethoxyethylidene)triphenylphosphorane to afford **116**. The enoate **116** was elaborated into the 1,3-benzothiazolesulfone **117** by standard transformations, prior to the cleavage of the Boc amide protecting group. The free amine **118** underwent an amidation with the TBS-protected (2*Z*,4*S*)-4-hydroxy-2-butenoic acid **12b** to give **119**. The modified Julia olefination of the aldehyde **73** with the anion derived from **119** proceeded with a high *E*-selectivity to generate the mixed cyclic ketal **120**. Finally, the removal of the C-4’ silyl protecting group, acylation of the resultant alcohol, removal of the C-4 silyl protecting group, and ketal hydrolysis generated FR901464 (**1**).

#### Fragment coupling via olefin cross-metathesis

**Scheme 20:**
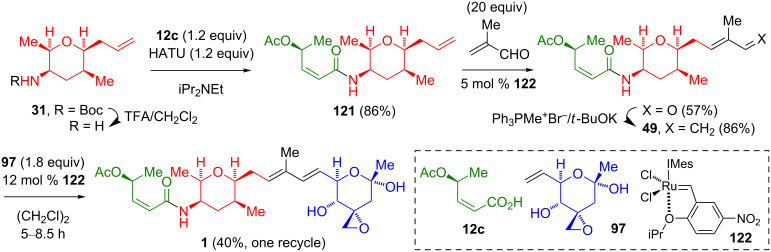
Fragment coupling via cross-metathesis by Koide.

Koide’s group was the first to demonstrate the use of a Ru-catalyzed olefin cross-metathesis for the generation of the dienyl segment joining the two tetrahydropyranyl segments (Scheme 20) [12–13]. The cleavage of the Boc amide of **31** and the eventual amidation with (2*Z*,4*S*)-4-acetoxy-2-butenoic acid (**12c**) gave **121**. The construction of the diene **49** involved cross-metathesis with an excess of methacrolein, catalyzed by **122**, followed by a Wittig olefination. The union of the diene **49** with 1.8 equivalents of the vinyl tetrahydropyran **97** was achieved with the Ru catalyst **122**. One recycle of the recovered starting material from this reaction gave FR901464 (**1**) in a combined yield of 40%. This reaction needed to be done at room temperature due to the lability of the hemiketal **97**. The use of Grubbs’ 2nd generation catalyst or the Grubbs–Hoveyda catalyst also gave **1**, albeit in a diminished yield (12–13%).

**Scheme 21:**
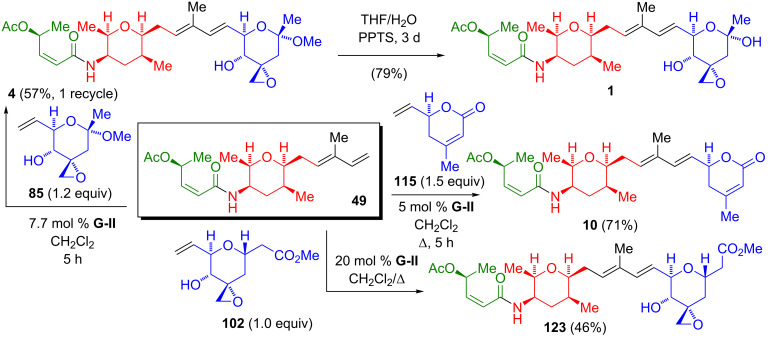
The Ghosh synthesis of spliceostatin A (**4**), FR901464 (**1**), spliceostatin E (**10**), and thailanstatin methyl ester (**123**).

The Ghosh synthesis of spliceostatin A (**4**), FR901464 (**1**) [15–16], spliceostatin E (**10**) [41], and thailanstatin A methyl ester (**123**) [31] used the cross-metathesis strategy for the coupling of the diene **49** with **85**, **115**, and **102**, respectively (Scheme 21). In order to avoid the decomposition problems encountered by Koide, the mixed cyclic ketal **85** was used as a coupling partner for the preparation of spliceostatin A (**4**). Due to this change, Grubbs’ second-generation catalyst, at a lower loading and elevated reaction temperatures, could be used since **4** was not labile under these thermal conditions. Ketal **4** underwent a hydrolysis to **1** under acidic conditions. The yields for the cross-metathesis coupling were generally higher when using an excess of **85** or **115**, as compared to the cross-metathesis of **102**, which also used a relatively high catalyst loading (20 mol % for 46% yield). While these authors did not report on the hydrolysis of **123** to prepare thailanstatin A (**7**), they found that the methyl ester **123** (IC_50_ ≈ 0.4 μM) is nearly equipotent with **7** (IC_50_ ≈ 0.65 μM).

#### Fragment coupling via Wittig olefination, cross-metathesis, and Julia olefination

**Scheme 22:**
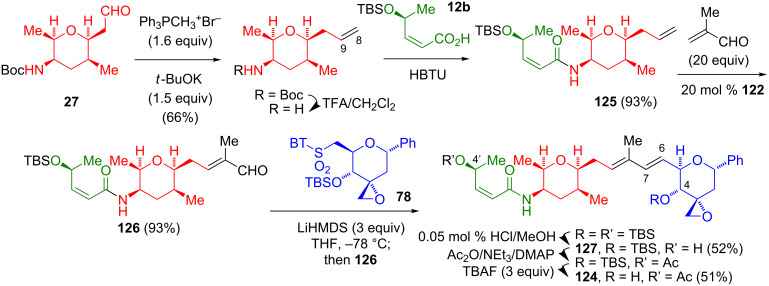
Arisawa synthesis of a C-1-phenyl analog of FR901464 (**1**).

In their recent synthesis of a 1-phenyl analog (**124**) of FR901464 (**1**), Arisawa’s group utilized a combination of previously reported methodologies (Scheme 22) [35]. Thus, the (all-*cis*)-2,3,5,6-tetrasubstituted aldehyde **27** was constructed according to Kitahara’s protocol [8–9]. The Wittig methenylation of **27**, followed by the cleavage of the Boc protecting group and the amidation with **12b** afforded the fragment **125**. The cross-metathesis of **125** with an excess of methacrolein in the presence of the Ru catalyst **122** gave the aldehyde **126**. This coupling is analogous to Koide’s cross-metathesis of **121** (cf. Scheme 20). A modified Julia olefination of **126** with the anion generated from the sulfone **78**, followed by the cleavage of the 4’-TBS protecting group gave **127** in an acceptable yield (52%). A protecting-group adjustment finalized the synthesis of **124**. Notably, these authors found that switching the sulfone and aldehyde functionalities, i.e., an olefination of the aldehyde generated by the oxidation of **77** with Kitahara’s sulfone **119**, proceeded less efficiently (22% yield). In a subsequent assay for repressed cell proliferation against the human prostate cancer cell lines LNCaP, LNCaP95, and CWR22Rv, these authors reported IC_50_ values of 63, 175, and 93 nM, respectively, for **124**. These can be compared to the IC_50_ values of 1.3, 1.0, and 0.36 nM, respectively, for spliceostatin A (**4**) against the same cell lines.

#### Fragment union via Pd-catalyzed sp^2^–sp^2^ coupling

**Scheme 23:**
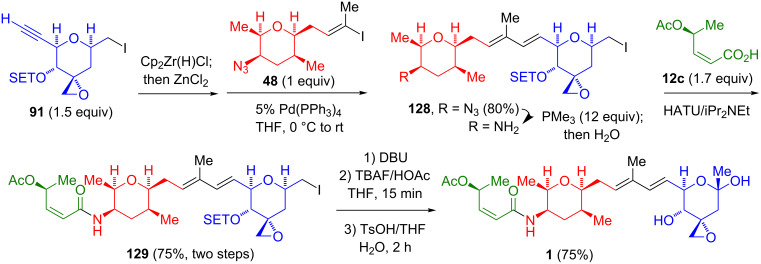
Jacobsen fragment coupling by a Pd-catalyzed Negishi coupling.

A Negishi coupling reaction [42] was used in the Jacobsen synthesis of FR901464 (**1**, Scheme 23) [17–18]. The hydrozirconation of **91**, followed by a transmetalation provided a vinylzinc reagent that was coupled with **48** to afford **128**, for which only the *E*-stereoisomer was observed. Notably, the Negishi conditions were tolerant to the azide present in **48** and the oxirane and 1° iodoalkane present in **91**. The subsequent reduction of the azide and the amidation with **12c** afforded **129**. The synthesis was completed by the elimination of the 1° iodide, silyl ether cleavage, and hydration of the exocyclic enol ether. Of all the syntheses of FR901464 (**1**), the coupling of **91** with **48** is the most efficient sequence (6 steps, 45% yield).

**Scheme 24:**
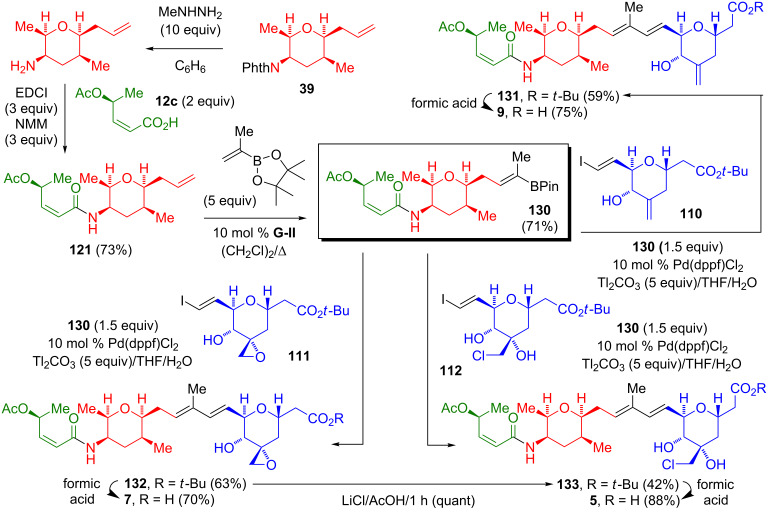
Nicolaou syntheses of thailanstatin A and B (**7** and **5**) and spliceostatin D (**9**) via a Pd-catalyzed Suzuki–Miyaura coupling.

Nicolaou utilized the sequential application of a cross-metathesis and a Suzuki–Miyaura coupling in the syntheses of thailanstatin A and B (**7** and **5**) and spliceostatin D (**9**, Scheme 24) [24–25]. To this end, the methylhydrazinolysis of the phthalimide **39** and the amide formation with **12c** yielded **121**. The cross-metathesis of **121** with a five-fold excess of isopropenylboronic acid pinacol ester afforded the lynchpin vinylborane **130**. A Pd-catalyzed Suzuki–Miyaura coupling of **130** with the vinyl iodide **110**, **111**, or **112** gave the *tert*-butyl ester **131**, **132**, or **133** in a moderate yield (42–63%). The hydrolysis of the *tert*-butyl esters with formic acid gave the carboxylic acid **9**, **7**, or **5**. Additionally, the treatment of the thailanstatin A *tert*-butyl ester with lithium chloride generated the thailanstatin B *tert*-butyl ester.

**Scheme 25:**
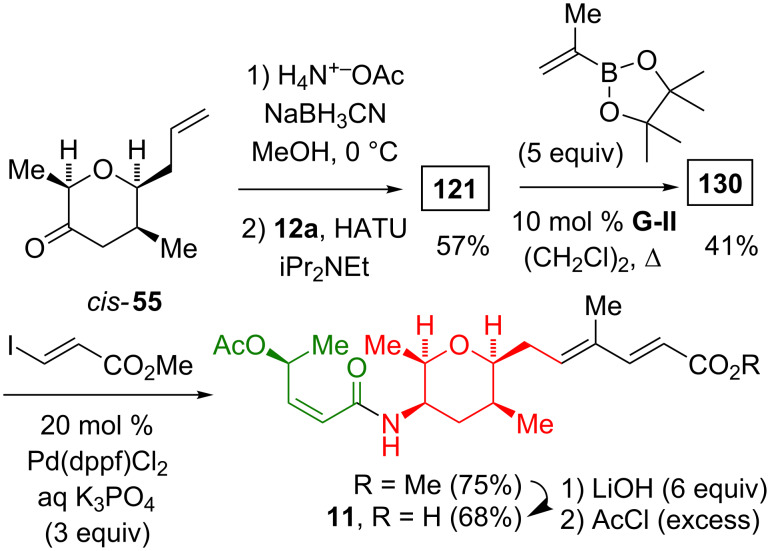
The Ghosh synthesis of spliceostatin G (**11**) via Suzuki–Miyaura coupling.

Ghosh used a similar cross-metathesis/Suzuki–Miyaura coupling sequence for the preparation of spliceostatin G (**11**, Scheme 25) [43]. The catalyst loading for the coupling of **130** with methyl (*E*)-3-iodoacrylate (20 mol %) was twice the amount used by Nicolaou (10 mol %).

## Conclusion

As delineated above, a wide variety of synthetic strategies has been employed to either introduce the stereocenters present in the spliceostatins/thailanstatins or to couple the subfragments of these molecules (Table 2). The most common route to the (2*Z*,4*S*)-4-acetoxy-2-butenoic acid fragment relies on the *cis*-reduction of 4-acetoxy-2-pentynoic acid; the most efficient of these routes utilizes a Noyori asymmetric reduction of an alkynyl ketone to produce the required stereocenter. Of the syntheses to date, Jacobsen’s use of an asymmetric Cr(III)-catalyzed cycloaddition stands as the most efficient route, in terms of synthetic steps and low catalyst loading, to the (all-*cis*)-2,3,5,6-tetrasubstituted tetrahydropyran and the C-1–C-6 tetrahydropyran fragments of FR901464 (**1**). The preparation of the C-1–C-5 tetrahydropyran fragment of the thailanstatins has, so far, utilized sugar-derived precursors. A variety of reactions have been utilized for union of these fragments via a diene segment. While a Ru-catalyzed cross-metathesis reaction was employed in numerous syntheses, the drawbacks of this strategy include the high catalyst loading and the excess of the olefin coupling partners. To date, the most efficient strategy for the union of the fragments relies on Pd-catalyzed sp^2^–sp^2^ couplings.

**Table 2:** Summary of the total syntheses.

target	PI	year	Reference	Scheme	no. of steps (LLS)	overall yield

FR901464 (**1**)	Jacobsen	2000	[17–18]	5, 14, 23	16	8.46%
FR901464 (**1**) 1st generation	Kitahara	2001	[8]	2, 10, 19	22	1.63%
FR901464 (**1**) 2nd generation	Kitahara	2006	[9]	2, 11, 19	23	3.18%
FR901464 (**1**)	Koide	2006	[12–13]	3, 15, 20	15	3.77%
spliceostatin A (**4**)	Ghosh	2013	[15–16]	3, 15, 20	11	6.61%
FR901464 (**1**)	Ghosh	2013	[15–16]	7, 13, 21	12	5.22%
spliceostatin E (**10**)	Ghosh	2014	[14]	7, 18, 21	9	8.24%
thailanstatin A (**7**)	Nicolaou	2016	[24]	4, 17, 24	12	2.23%
thailanstatin A methyl ester (**123**)	Ghosh	2018	[31]	8, 16, 21	15	0.54%
spliceostatin G (**11**)	Ghosh	2018	[43]	9, 25	15	2.31%
spliceostatin D (**9**)	Nicolaou	2018	[25]	4, 17, 24	12	2.24%
thailanstatin B (**5**)	Nicolaou	2018	[25]	4, 17, 24	12	1.87%
spliceostatin A analog	Arisawa	2019	[34]	2, 12, 22	18	3.99%
